# MicroRNA-153 is a prognostic marker and inhibits cell migration and invasion by targeting SNAI1 in human pancreatic ductal adenocarcinoma

**DOI:** 10.3892/or.2022.8444

**Published:** 2022-11-14

**Authors:** Zhenghai Bai, Jiangli Sun, Xiaobo Wang, Hai Wang, Honghong Pei, Zhengliang Zhang

Oncol Rep 34: 595–602, 2015; DOI: 10.3892/or.2015.4051

Subsequently to the publication of the above article, a concerned reader drew to the Editors' attention that the cell invasion and migration assay data shown in Fig. 3B and D were strikingly similar to data appearing in different form in other articles by different authors. Owing to the fact that these contentious data in the above article had already been published elsewhere, or were already under consideration for publication, prior to its submission to *Oncology Reports*, the Editor has decided that this paper should be retracted from the Journal. After having been in contact with the authors, they agreed with the decision to retract the paper. The Editor apologizes to the readership for any inconvenience caused.

